# Biomaterials-based phototherapy for bacterial infections

**DOI:** 10.3389/fphar.2024.1513850

**Published:** 2024-12-04

**Authors:** Guangzhi Wu, Zhuo Xu, Yue Yu, Minglei Zhang, Shuaishuai Wang, Shuo Duan, Xilin Liu

**Affiliations:** ^1^ Department of Hand & Foot Surgery, China-Japan Union Hospital of Jilin University, Changchun, China; ^2^ Department of Rehabilitation, China-Japan Union Hospital of Jilin University, Changchun, China; ^3^ Department of Infectious Diseases, Orthopedic Center, The First Hospital of Jilin University, Jilin University, Changchun, China; ^4^ Department of Orthopedics, China-Japan Union Hospital of Jilin University, Changchun, China

**Keywords:** antibacterial, phototherapy, photodynamic therapy, photothermal therapy, photoinitiator

## Abstract

Bacterial infections and antibiotic resistance are global health problems, and current treatments for bacterial infections still rely on the use of antibiotics. Phototherapy based on the use of a photosensitizer has high efficiency, a broad spectrum, strong selectivity, does not easily induce drug resistance, and is expected to become an effective strategy for the treatment of bacterial infections, particularly drug-resistant infections. This article reviews antimicrobial strategies of phototherapy based on photosensitizers, including photodynamic therapy (PDT), photothermal therapy (PTT), and their combination. These methods have significant application potential in combating multi-drug-resistant bacterial and biofilm infections, providing an alternative to traditional antibiotics and chemical antibacterial agents.

## 1 Introduction

Bacterial infections pose a significant public health threat worldwide, especially with the rapid rise of antibiotic-resistant strains (Kim et al., 2022). The widespread and often irrational use of antibiotics has contributed to the emergence and spread of resistant pathogens, such as methicillin-resistant *Staphylococcus aureus* (*MRSA*), vancomycin-resistant enterococci, and multidrug-resistant Gram-negative bacteria (Dehbanipour and Ghalavand, 2022). Traditional antimicrobial strategies are facing unprecedented challenges due to the increasing prevalence of drug-resistant bacterial infections (Macnair et al., 2024). According to the World Health Organization (WHO), antibiotic resistance is responsible for approximately 700,000 deaths annually, with this figure projected to rise to 10 million by 2050 (Umarje and Banerjee, 2023). Therefore, developing effective antimicrobial strategies that are not prone to resistance is a critical priority in infection control.

Significant advancements have been made in combating antimicrobial resistance, particularly in fields such as nanotechnology, targeted drug delivery, gene editing, immunotherapy, and phototherapy (Bucataru and Ciobanasu, 2024). Nanomaterials, including metallic nanoparticles and carbon nanotubes (CNTs), exhibit broad-spectrum antibacterial activity by directly damaging bacterial membranes and generating reactive oxygen species (ROS), with the added advantage of being less likely to induce resistance (Maksimova and Zorina, 2024). However, concerns remain regarding their potential toxicity, biocompatibility, and metabolic pathways, which could pose long-term safety risks (Chausov et al., 2022; Roesner et al., 2023; You et al., 2024). Although targeted delivery systems precisely direct antimicrobial agents to infection sites and enhance therapeutic efficacy, their high cost of production and the complex distribution and clearance mechanisms of nanocarriers require further research (Qu and Zhu, 2023; Yu S. et al., 2024). Gene editing, which targets bacterial resistance genes, offers a highly specific approach but is still in its early stages, with challenges related to accuracy, off-target effects, and safety (Devi et al., 2023; Li X. et al., 2023). Immunotherapy also enhances the host’s ability to clear infections; however, it may trigger excessive immune responses or autoimmune diseases, limiting its application (Pang X. et al., 2023; Wang H. et al., 2023).

Phototherapy-based antimicrobial strategies have the potential to address many of these challenges while avoiding the development of bacterial resistance (Sedighi et al., 2024; Yu B. et al., 2024). In recent years, photosensitizer-based therapies have gained significant attention. Photosensitizers used in antimicrobial applications can be classified into two main types: photosensitizers and photothermal agents (Yan S. et al., 2023; Zheng and Xiao, 2022). Photosensitizers mainly produce reactive oxygen species through photochemical reactions to achieve photodynamic therapy (PDT), while photothermal agents produce local high temperature through photothermal effect for photothermal therapy (PTT). These agents function through distinct mechanisms upon exposure to light, leading to bacterial cell death (Ruan et al., 2023). Photothermal agents absorb light energy and convert it into heat, raising local temperatures to levels (40°C–60°C) that are sufficient to denature bacterial proteins, disrupt cell membranes, and ultimately cause bacterial death (Lim et al., 2023). This approach, known as PTT, is a physical method of bacterial eradication. In contrast, photosensitizers, the core component of PDT, absorb light energy and transfer it to surrounding oxygen molecules, generating singlet oxygen or other ROS (Xiao et al., 2024). These reactive species possess strong oxidative properties, enabling them to damage bacterial cell membranes, cell walls, proteins, and nucleic acids, thereby leading to cell death (Ruan et al., 2023).

These two phototherapy approaches have shown significant promise in treating multidrug-resistant bacterial infections and biofilm-associated infections. This review focuses on photoinitiator-based antimicrobial strategies, particularly the classification of various photosensitizers and photothermal agents, and their applications in combating bacterial infections. We explore the use of metalloporphyrin-based photosensitizers, organic small-molecule photosensitizers, polymer-based photosensitizers, nanocomposite photosensitizers, metallic nanomaterials, carbon-based materials, and organic molecular photothermal agents. Finally, the synergistic effects of combining PDT and PTT will be discussed, offering new perspectives and future directions for antimicrobial therapies.

## 2 Photodynamic therapy for fighting bacterial infections

PDT uses photosensitizers that generate ROS under light irradiation to kill bacteria (Xiao et al., 2024). This approach has garnered significant attention in antimicrobial therapy due to its high efficiency, broad-spectrum activity, and limited tendency to induce drug resistance.

### 2.1 Metalloporphyrin-based photosensitizers for treating bacterial infections

**FIGURE 1:**
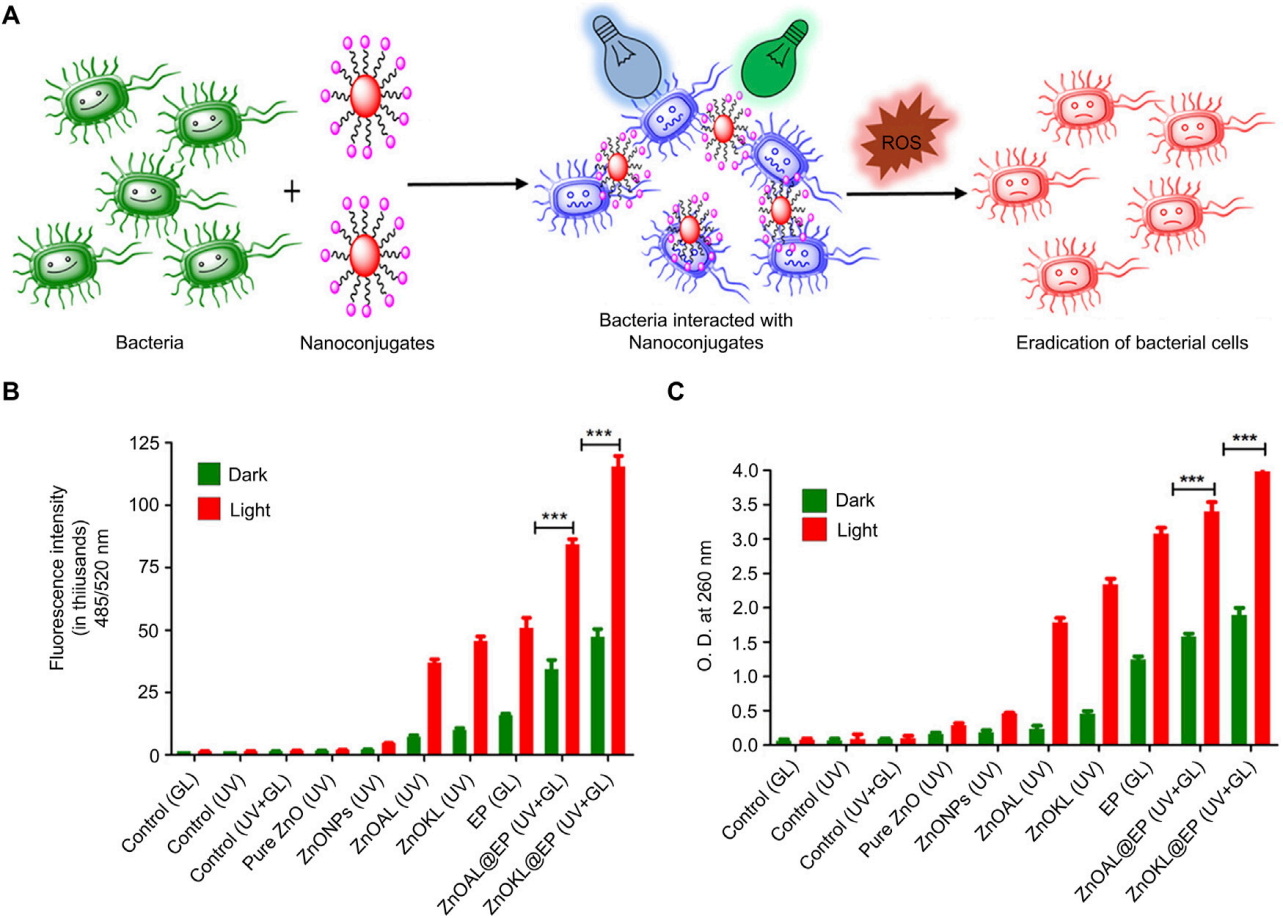
Antibacterial mechanism and effect of nanoconjugates (Pujari et al., 2024) **(A)** Schematic of bacterial eradication **(B)** ROS generation studies of DCFH-DA-mediated nanoconjugates **(C)** Nucleic acid release studies of nanoconjugates Data are presented as the mean ± SD (*n* = 3 in each group). Statistical data are represented as mean ± SD (*n* = 3; *P < 0.05, **P < 0.01, ***P < 0.001, and ****P < 0.0001; NS represents no significant difference). Copyright ^©^ 2024, American Chemical Society.

Metalloporphyrin-based photosensitizers are porphyrin ring compounds in which the introduction of metal ions (such as zinc, aluminum, or ruthenium) enhances their photosensitizing properties (Hlabangwane et al., 2023). Pujari et al. coupled metal porphyrins to lignin-based zinc oxide to develop hydrophilic nanoconjugates that showed significantly improved PDT efficiency when treating bacterial infections under dual light irradiation (Figure 1A) (Pujari et al., 2024). The nanoconjugates showed the highest fluorescence intensity when exposed to dual light (UV and green light) and exhibited high bactericidal activity due to increased ROS generation capacity (Figure 1B). In addition, the results of nucleic acid leakage experiments showed that the nanoconjugates resulted in more DNA leakage under dual light irradiation compared to single light irradiation, further supporting their ROS generation efficiency. The overall findings showed that the nanoconjugates had a destructive effect on microbial cells through the ROS produced by PDT, thereby achieving an effective antibacterial effect (Figure 1C).

These photosensitizers absorb light, particularly in the visible range (400–700 nm), making them highly effective for PDT applications (Abbas et al., 2023). Upon light exposure, metalloporphyrins transfer energy to surrounding oxygen molecules, producing singlet oxygen and other ROS (Bi et al., 2023). These reactive species have strong oxidizing properties and are capable of destroying bacterial cell walls, cell membranes, and critical intracellular components, including proteins and nucleic acids, to achieve an antimicrobial effect (Kumar et al., 2023).

Metalloporphyrin photosensitizers demonstrate broad-spectrum antimicrobial activity, especially against drug-resistant strains (Michalska et al., 2022). Zinc (II) porphyrin (ZnP), for instance, has been proven to be effective in killing bacteria, such as *MRSA*, *Escherichia coli*, and *Pseudomonas aeruginosa*. Studies have shown that ZnP, when irradiated, generates ROS that significantly damage bacterial cell membranes, leading to the leakage of intracellular contents and subsequent bacterial death (Nejad et al., 2022).

Biofilm infections, characterized by a complex extracellular polymer matrix that shields bacteria from antibiotics and immune system attacks, pose a major challenge in chronic infections. Metalloporphyrin photosensitizers offer promising solutions against biofilms (Pucelik et al., 2024). Aluminum phthalocyanine chloride (AlPcCl), for example, has demonstrated excellent photosensitizing efficacy against *Pseudomonas aeruginosa* biofilms. Upon light exposure, AlPcCl penetrates the biofilm and generates ROS, killing greater than 90% of the embedded bacteria (Souza et al., 2022).

While the antimicrobial efficacy of metalloporphyrin photosensitizers has been well documented in both laboratory and animal models, their clinical application remains in the early stages (Zhang et al., 2023). Preclinical studies suggest promising therapeutic outcomes for superficial bacterial infections, such as skin infections, postoperative wound infections, and periodontal diseases (Castillo et al., 2024). Notably, zinc porphyrin-based PDT significantly reduced bacterial loads and accelerated wound healing in *MRSA*-infected skin models.

However, some limitations must be addressed. Metalloporphyrin photosensitizers typically require visible light for activation, limiting their penetration into deeper tissues and reducing their efficacy in treating deep-seated infections. Additionally, the success of PDT depends on adequate oxygen supply to tissues, and hypoxic environments, such as those of deep infections or abscesses, may reduce treatment effectiveness. Though metalloporphyrin photosensitizers have demonstrated good safety profiles in *in vitro* and animal studies, their long-term safety and metabolic pathways in humans require further research.

Future efforts should focus on molecular modification and functional design to enhance the light absorption properties and ROS generation by metalloporphyrin photosensitizers. Combining these photosensitizers with other antimicrobial technologies could further improve their efficacy, positioning metalloporphyrin-based PDT as a valuable tool in fighting drug-resistant bacterial infections.

### 2.2 Organic small-molecule photosensitizers for treating bacterial infections

Organic small-molecule photosensitizers are compounds with relatively low molecular weight and diverse structures, offering significant advantages in antibacterial therapy (Atac et al., 2024). Their structural versatility and efficient photosensitization properties make them ideal candidates for antimicrobial applications. Due to their simpler molecular structures and ease of synthesis, organic small-molecule photosensitizers can be functionally modified to enhance their photosensitizing performance, broaden their light absorption range, and improve their biocompatibility (Gill et al., 2022). These properties contribute to their effectiveness against a variety of pathogens, including Gram-positive bacteria, Gram-negative bacteria, and fungi, with particular success in combating drug-resistant strains like *MRSA* and VRE (Gong et al., 2024).

**FIGURE 2:**
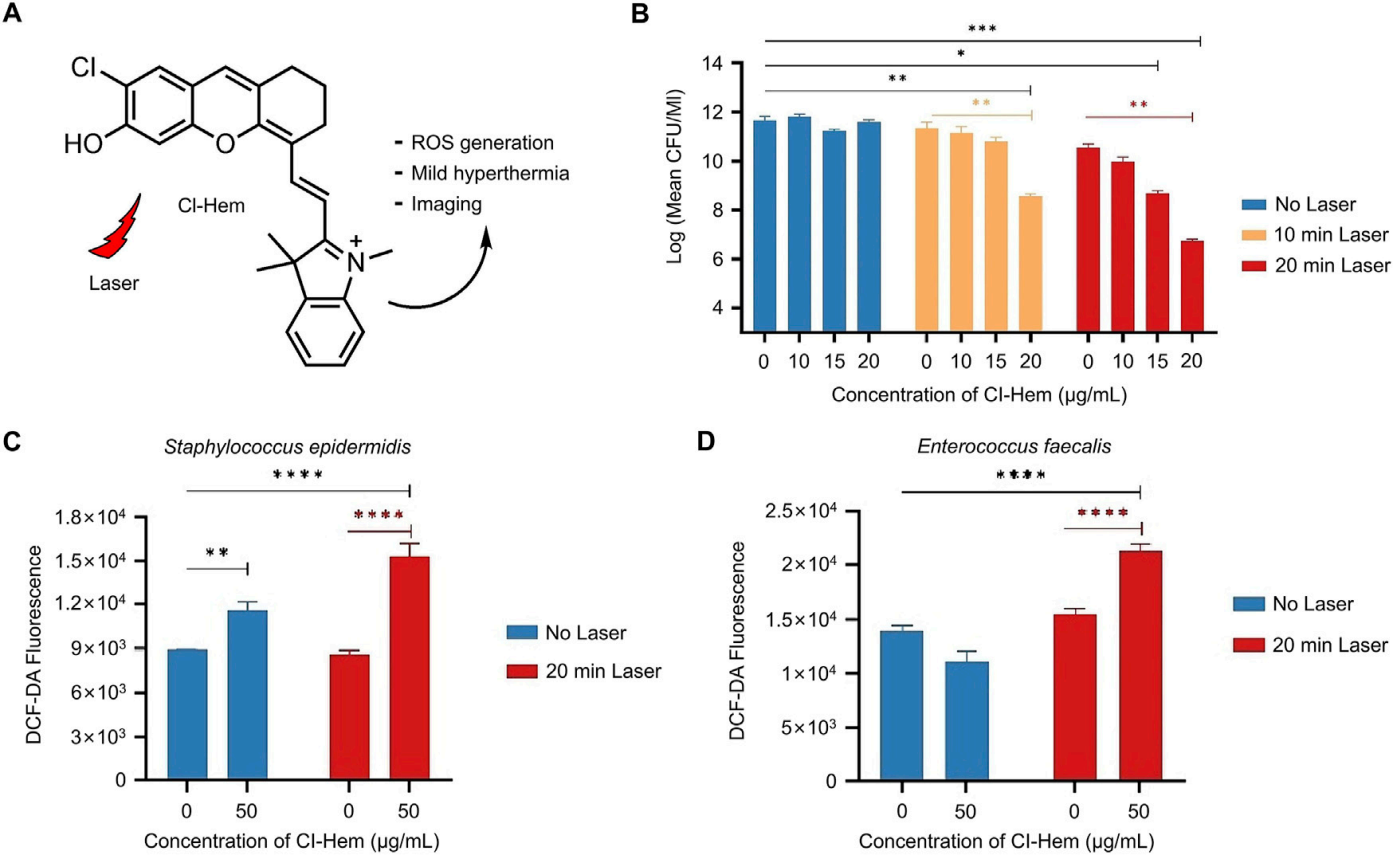
Antibacterial mechanism and effect of Cl-Hem (Atac et al., 2024) **(A)** Molecular structure of Cl-Hem **(B)** Dose-dependent antibacterial effect of Cl-Hem on plankcells of *Staphylococcus epidermidis* with/without laser irradiation **(C)** Fluorescence intensity of DGF-DA in Gram-positive cells treated with Cl-Hem (0 or 50 μg/mL) **(D)** Fluorescence intensity of DCF-DA in Cl-HEM-treated Gram-negative bacteria. Data are presented as the mean ± SD (*n* = 3 in each group). Statistical data are represented as mean ± SD (*n* = 3; *P < 0.05, **P < 0.01, ***P < 0.001, and ****P < 0.0001; NS represents no significant difference). Copyright ^©^ 2024 Elsevier B.V.

Atac et al. studied Cl-Hem, an organic small-molecule photosensitizer, and explored its bactericidal effect (Figure 2A) (Atac et al., 2024). Dose-dependent antibacterial effects were verified in plankcells and biofilms of Gram-positive bacteria with and without 640-nm laser irradiation (Figure 2B). Based on the characteristic green fluorescence of the ROS sensor (Figures 2C, D), a significant increase (73%) in *Streptococcus* epidermalis killing was observed at 50 μg/mL of Cl-Hem 20 min after laser irradiation (*p* < 0.0001). Interestingly, this dose of Cl-Hem also resulted in a 30% increase in ROS in the absence of laser irradiation (*p* = 0.0016). However, no antibacterial effect was observed without laser irradiation, indicating that photoinduced toxicity was the main source of antibacterial activity. Although Cl-Hem produced some amount of ROS in the absence of light, its concentration and reactivity were not sufficient to cause significant bacterial damage. In contrast, ROS production under light exposure was higher and more active enough to trigger the destruction of bacterial cell membranes and cellular structures to achieve effective bactericidal effects.

One of the key advantages of certain organic small-molecule photosensitizers is their absorption capacity in the near-infrared (NIR) range, which allows deeper tissue penetration and the ability to treat infections that reside in deeper tissue layers (Hao et al., 2024). For example, indocyanine green (ICG), a widely used cyanine dye, absorbs light at approximately 808 nm and has been shown to be particularly effective in PDT for deep tissue infections (Liu X. et al., 2022).

The photosensitization mechanism of these organic small molecules typically involves excitation under specific wavelengths of light, transitioning them from a singlet state to a triplet state where they transfer energy to oxygen molecules. This energy transfer produces ROS, such as singlet oxygen, which cause oxidative damage to bacterial cell membranes, proteins, and DNA, eventually leading to bacterial death.

ICG is one of the most extensively studied cyanine dye photosensitizers due to its excellent NIRabsorption properties and strong tissue penetration. Studies have demonstrated ICG’s ability to effectively kill a variety of pathogenic bacteria, including *MRSA*, *Escherichia coli* (Xiong et al., 2024), and *Pseudomonas aeruginosa*, under light irradiation. For example, in one study, ICG significantly promoted wound healing in *MRSA*-infected wounds following near-infrared irradiation. This makes ICG particularly suitable for treating recalcitrant infections, such as infections following skin injuries and post-surgical infections.

Rhodamine B, a photosensitizer commonly used for cell labeling and fluorescent probes, has shown great potential for antimicrobial PDT. Studies indicate that rhodamine-type photosensitizers generate ROS under light irradiation, effectively killing both Gram-positive and Gram-negative bacteria (Yan Y. et al., 2023). For instance, in one experiment, rhodamine B demonstrated a bactericidal rate of over 90% against *MRSA* and 85% against *Pseudomonas aeruginosa*, while also inhibiting biofilm formation (Xue et al., 2023). Its straightforward structure, high stability, and ease of synthesis and modification make rhodamine B a promising candidate for multifunctional antimicrobial materials.

Coumarin-based photosensitizers are known for their wide light absorption range and ability to generate ROS in the UV-visible range (Xiong et al., 2024). Although less frequently studied in antimicrobial PDT, coumarin photosensitizers have been shown to kill Gram-positive and Gram-negative bacteria after photosensitization. In one study, coumarin photosensitizers effectively disrupted the cell membranes of *Escherichia coli* and *MRSA*, increasing cell membrane permeability and accelerating bacterial death (Ma M. et al., 2024).

Organic small molecule photosensitizers have broad-spectrum antimicrobial activity, particularly against drug-resistant strains and biofilm-associated infections. However, certain limitations must be addressed. These include the need for specific light sources, potential problems with photostability, and phototoxicity concerns. To overcome these challenges, future research should focus on modifying the molecular structure of these photosensitizers to improve their properties, incorporating nanotechnology, and developing combination therapy strategies.

Through continued innovation and optimization, organic small-molecule photosensitizers hold promise for becoming more effective tools in combating bacterial infections, particularly in clinical settings.

### 2.3 Polymer-based photosensitizers for treating bacterial infections

Polymer-based photosensitizers are composite materials that integrate photosensitizers with polymer matrices, typically through covalent bonds or non-covalent interactions (Ahmetali et al., 2023). The polymer matrix, which may be composed of natural or synthetic materials, enhances the stability, biocompatibility, and sustained release of the photosensitizer at the infection site (Bryaskova et al., 2023). This design reduces the non-specific aggregation of photosensitizers *in vivo*, ensuring more targeted and efficient antimicrobial effects. The underlying mechanism remains consistent with that of traditional PDT, where ROS are generated upon light irradiation, leading to bacterial cell membrane damage and cell death (Chen et al., 2024).

Common polymer materials used in such composites include polyethylene glycol (PEG), chitosan, and polylactic-co-glycolic acid (PLGA). These polymers not only improve the biological stability of the photosensitizers but also allow for controlled release, making them highly effective in clinical applications (Elian et al., 2024).

Incorporating porphyrin-based photosensitizers into polymer matrices significantly improves their biostability and antimicrobial efficacy *in vivo*. For example, porphyrin photosensitizers combined with PEG have demonstrated enhanced photosensitization and increased antimicrobial activity under light irradiation (Hino et al., 2024). Studies have shown that such composites effectively kill *MRSA* and *Escherichia coli* with a sterilization rate exceeding 95% (Jiao et al., 2022). Additionally, these polymer-porphyrin complexes have been developed into antimicrobial coatings, such as those used in wound dressings and on medical implant surfaces. These coatings continuously release ROS, preventing bacterial infections at wound sites (Liang et al., 2022).

Chitosan, a naturally derived polymer with intrinsic antimicrobial properties and excellent biocompatibility, has been widely applied in wound dressings, biomedical implants, and antimicrobial coatings (Ma Z. et al., 2024). When combined with photosensitizers, chitosan significantly enhances their antimicrobial effect while also providing long-lasting antibacterial activity. Chitosan-based photosensitizers have shown great promise, particularly in antimicrobial wound dressings (Manathanath et al., 2024). Research has demonstrated that the combination of chitosan with porphyrin photosensitizers improves bactericidal efficiency at infection sites under light exposure, showing potent effects against drug-resistant bacteria, including *MRSA* (Qian et al., 2024).

Cyanine dyes, such as ICG, have gained widespread attention for their excellent light absorption properties in the NIR region (Wang et al., 2022). When combined with polymeric materials, cyanine dye-based photosensitizers exhibit enhanced stability and antimicrobial efficacy. For example, composites of ICG and PEG significantly improve the production of ROS under NIR light, effectively killing *MRSA* and *E. coli* in deep tissue infections. These composites are particularly advantageous for treating infections that are difficult to cure by conventional antimicrobial methods (Xu et al., 2022).

Titanium dioxide (TiO₂) is a well-known photocatalytic material that is often used as an antimicrobial agent when combined with polymers (Tsai et al., 2024). Polymer-based TiO₂ photosensitizers are not only effective in antimicrobial coatings but also widely used in water treatment and environmental applications. For example, PLGA combined with TiO₂ has demonstrated high efficacy in killing environmental bacteria, such as *E. coli* and *MRSA,* upon light irradiation (Zhou et al., 2024).

The application of polymer-based photosensitizers has great potential, especially in combating biofilm infections and developing long-lasting antimicrobial materials. Embedding photosensitizers into polymer matrices greatly enhances their biocompatibility and stability, ensuring efficient antimicrobial activity *in vivo*.

However, certain challenges remain. These include limited light penetration, potential material degradation, and the complexity of synthesizing polymer-based photosensitizers. Future research should focus on optimizing the combination of photosensitizers with polymer matrices to improve antimicrobial efficacy. Developing low-cost, biodegradable, and efficient polymer-based photosensitizers could further promote their use in clinical settings.

## 3 Photothermal therapy for fighting bacterial infections

PTT is a treatment modality that converts light energy into heat energy through photothermal agents, creating localized high temperatures to kill bacteria. Unlike PDT, PTT does not rely on oxygen to exert its effects, allowing it to maintain efficacy even in hypoxic environments, such as biofilm-related or deep tissue infections. PTT utilizes various types of photothermal agents, including metallic nanomaterials, carbon-based materials, polymer-based materials, and organic molecular photothermal agents, each of which has distinct antimicrobial mechanisms.

### 3.1 Metallic nanomaterial-based photothermal agents for treating bacterial infections

Metallic nanomaterials have emerged as a research hotspot due to their excellent photothermal conversion efficiency, biocompatibility, and potent bactericidal effects against a wide range of pathogens (Ahmad and Ansari, 2022). These agents absorb specific wavelengths of light (typically in the NIR range) and convert this energy into heat, raising local temperatures to 40°C–60°C, which disrupts bacterial cell membranes, walls, and proteins, ultimately leading to cell death (Amarasekara et al., 2024). Common metallic nanomaterials used in PTT include gold, silver, copper, palladium, and ruthenium nanoparticles (Dediu et al., 2023).

**FIGURE 3:**
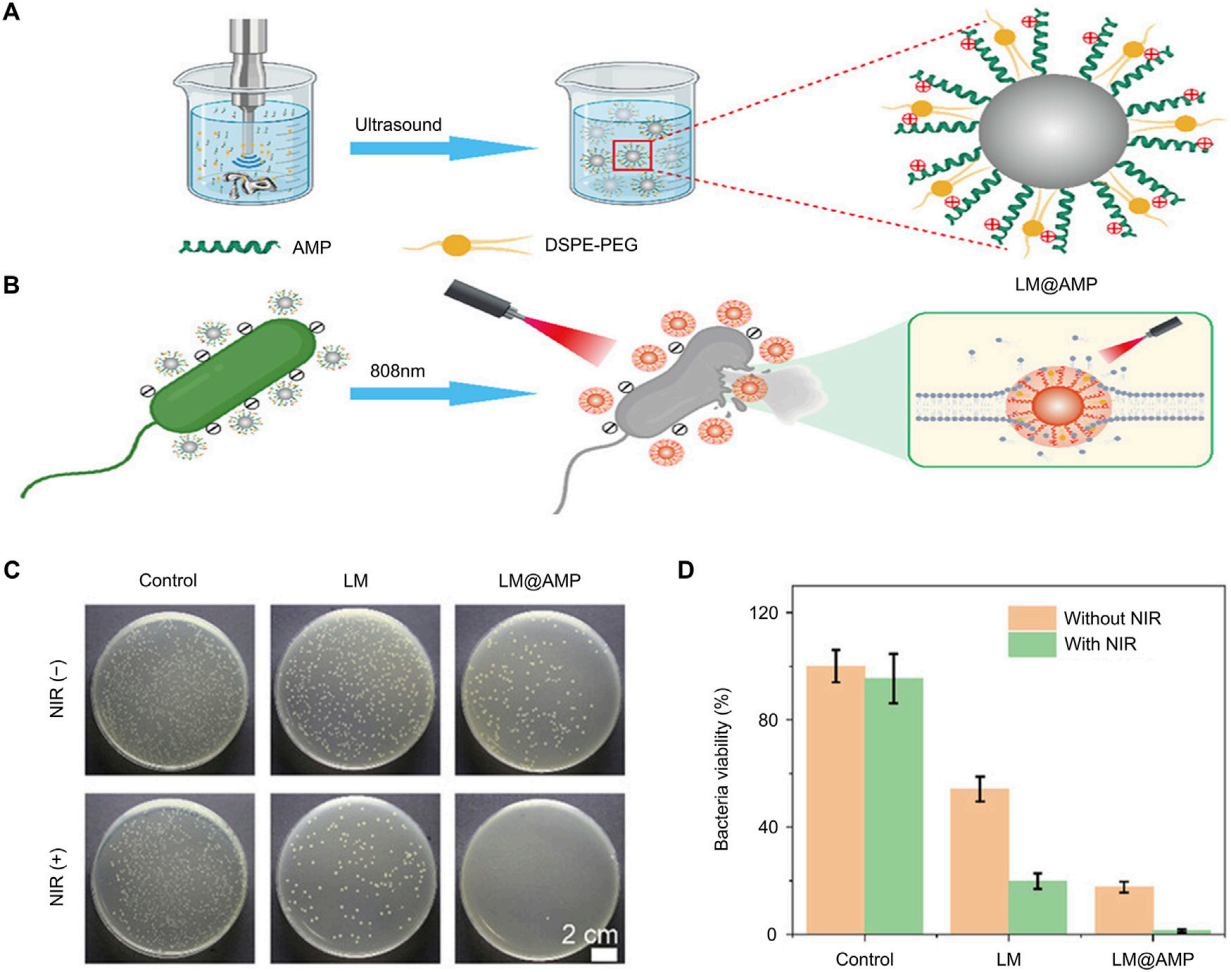
Antibacterial mechanism and effect of LM@AMP (Wang B. et al., 2024) **(A)** Schematic representation of photothermal LM@AMP nanoparticle preparation **(B)** Photothermal killing of bacteria **(C)** Photographs of AGAR plates subjected to various treatments **(D)** Quantitative data with various treatments Statistical data are represented as mean ± SD (*n* = 3).

Despite its many advantages, the clinical applicability of PTT as the sole sterilization strategy is hampered by the necessity of higher temperatures that can potentially harm healthy tissues (Ding et al., 2022). To overcome this challenge, Wang et al. introduced antimicrobial peptides (AMPs) to modify the surface of gallium-based liquid metal (LM) nanoantibacterial agents, thereby enhancing their photothermal antibacterial effects at lower temperatures (Figures 3A, B) (Wang B. et al., 2024). Although LM nanoparticles exhibit some antibacterial properties, their bactericidal effect is relatively limited. The researchers demonstrated a notable reduction in the number of bacteria after 808 nm NIR laser irradiation, thereby showing that their method significantly enhanced the antibacterial ability of the material system (Figures 3C, D).

Gold nanoparticles (AuNPs), particularly gold nanorods (GNRs), are widely used in antimicrobial research due to their efficient photothermal conversion at NIR wavelengths (808 nm) (Doveri et al., 2023). Under light irradiation, GNRs rapidly elevate the temperature to 60 °C, which is sufficient to disrupt bacterial cell membranes (Gu et al., 2023). Studies have demonstrated that GNRs achieve over 95% bactericidal rates against *MRSA* and *Escherichia coli* (Hajfathalian, 2022). In combination with antibiotics, GNRs significantly enhance the efficacy of antimicrobial treatments against multidrug-resistant bacteria like *MRSA* (Hajfathalian, 2024). Additionally, AuNPs have been incorporated into medical dressings to treat chronic wound infections, significantly accelerating wound healing (Ilhami et al., 2023).

Silver nanoparticles (AgNps) exhibit broad-spectrum antimicrobial properties in addition to their excellent photothermal effects. When exposed to light, AgNPs rapidly heat up and disrupt bacterial membranes (Joudeh et al., 2022). The release of silver ions (Ag+) further inhibits bacterial growth by interacting with bacterial proteins and DNA (Li X. et al., 2024). In one study, AgNPs successfully eradicated over 99% of *MRSA* when used for PTT (Lin et al., 2022). These nanoparticles are also used as nanocarriers for antibiotics, enhancing targeted delivery and antimicrobial efficacy (Ma et al., 2022). AgNP-coated medical devices, such as catheters and implants, prevent bacterial infections and biofilm formation through their combined photothermal and antimicrobial properties (Liu et al., 2023).

Copper nanoparticles have garnered attention for their cost-effectiveness and high photothermal conversion efficiency. CuNPs have demonstrated potent bactericidal effects against both Gram-positive and Gram-negative bacteria, including *MRSA* and *E. coli* (Mammari and Duval, 2023). In one study, CuNPs achieved a 97% killing rate of *MRSA* in biofilm environments. However, the biosafety of copper nanoparticles remains a concern, as excessive copper accumulation *in vivo* may trigger oxidative stress and damage host cells (Manivasagan et al., 2024).

Palladium and ruthenium nanoparticles are being explored for their multifunctional properties in antimicrobial applications (Miao et al., 2024). In addition to their photothermal effects, these nanoparticles catalyze the production of ROS, further enhancing their antimicrobial effect (Mutalik et al., 2022). Palladium nanoparticles have been shown to kill *Pseudomonas aeruginosa* and *MRSA* with broad-spectrum activity, demonstrating their potential in tackling resistant bacterial infections (Naskar and Kim, 2022).

Metallic nanomaterials have shown tremendous potential in addressing the challenge of bacterial resistance. Gold, silver, and copper nanoparticles, in particular, offer multiple mechanisms for combating bacterial infections, including direct photothermal damage and synergistic effects with other antimicrobial methods (Pang Q. et al., 2023; Sethulekshmi et al., 2022; Sharma and Arnusch, 2022). However, challenges such as potential toxicity, limited light penetration *in vivo*, and the metabolic pathways of these materials require further investigation. Future research should focus on optimizing nanoparticle design, developing intelligent delivery systems, and conducting clinical studies to assess the safety and efficacy of these photothermal agents. Metallic nanomaterials could become a critical tool in treating multidrug-resistant bacterial infections, especially those involving biofilms.

### 3.2 Carbon-based material photothermal agents for treating bacterial infections

Carbon-based materials possess unique structural and physicochemical properties that allow them to efficiently convert light energy into heat, thereby killing bacteria (Balou et al., 2022). Common carbon-based photothermal agents include graphene and its derivatives, CNTs, carbon quantum dots (CQDs), and fullerenes (Dediu et al., 2023).

**FIGURE 4:**
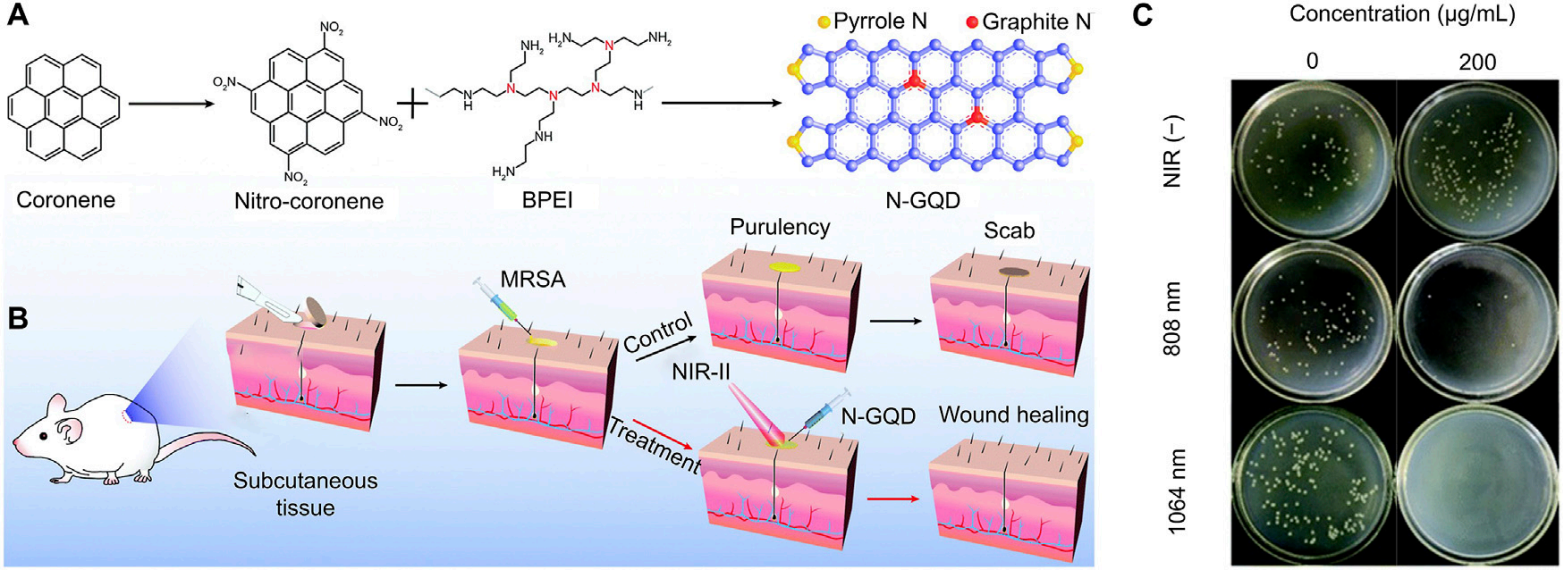
Antibacterial mechanism and effect of N-GQDs (Geng et al., 2022) **(A)** Preparation steps of N-GQDs **(B)** Schematic diagram of its application in the photothermal eradication of MDR bacterial infection **(C)** Representative culture images of colonies treated with various concentrations of N-GQD aqueous solutions.

Geng et al. synthesized high graphidic acid N-doped graphene quantum dots (N-GQD) with efficient NIR-II photothermal conversion properties for photothermal antibacterial therapy (Figures 4A, B) (Geng et al., 2022). The obtained N-GQDs showed strong NIR absorption in the range of 700–1,200 nm and achieved high photothermal conversion efficiencies of 77.8% and 50.4% at 808 and 1,064 nm, respectively. In the presence of 808 or 1,064 nm laser, N-GQD achieved excellent antibacterial and anti-biofilm activity against MDR bacteria (methicillin-resistant *MRSA*, *MRSA*) (Figure 4C). *In vivo* studies confirmed that hyperthermia generated by N-GQD plus NIR-II laser antagonized MDR bacterial infection and thereby significantly accelerated wound healing.

Graphene oxide (GO), with its two-dimensional structure and high photothermal conversion efficiency, is widely used in antimicrobial research. When exposed to NIR light (808 nm), GO generates localized high temperatures that kill bacteria (Chao et al., 2023). Additionally, GO itself possesses intrinsic antibacterial activity, physically disrupting bacterial cell membranes and inducing oxidative stress. Studies have shown that GO achieves a sterilization rate of 90% against *MRSA* and *E. coli* in PTT applications, and it penetrates and destroys biofilms, significantly enhancing the effectiveness of PTT (Chu et al., 2022).

Both single-walled and multi-walled CNTs (SWCNTs and MWCNTs) have high specific surface areas and light absorption capacities, making them effective in generating heat for PTT (Demirel et al., 2022). Under NIR light, CNTs raise local temperatures to 50°C–60°C, which is sufficient to kill pathogenic bacteria (Dong et al., 2023). CNTs may also be combined with other antimicrobial agents, such as AgNps or antibiotics, to form multifunctional composites that enhance their antimicrobial effects. In one study, SWCNTs effectively killed *MRSA* and *E. coli* under light exposure while also disrupting biofilm structures (Fang M. et al., 2023).

CQDs have gained significant attention in antimicrobial applications due to their nanometer size, excellent photothermal conversion efficiency, and biocompatibility (Feng et al., 2024). CQDs generate heat upon light absorption and also be designed to target specific bacterial infections. Studies have shown that CQDs are effective in killing *Pseudomonas aeruginosa* and *MRSA*, demonstrating their broad-spectrum antimicrobial activity. CQDs are also suitable for coating wound dressings and medical devices to prevent infections (Geng et al., 2022).

Fullerenes and their derivatives, with their unique cage-like structures, show potential in PDT (Hu et al., 2024a). Fullerenes efficiently generate heat under light irradiation and can be combined with photosensitizers to enhance PDT, creating a dual antimicrobial mechanism. In one study, fullerenes combined with AgNps effectively killed *MRSA* and *E. coli* under light exposure, demonstrating synergistic effects (Huo et al., 2024).

Carbon-based materials, such as graphene, CNTs, CQDs, and fullerenes, have shown great promise in antimicrobial PTT due to their efficient photothermal conversion properties and broad-spectrum activity (Jiang et al., 2024). However, challenges related to light penetration, biosafety, and the complexity of the material preparation process remain barriers to clinical translation (Lagos et al., 2023). Future research should focus on developing more efficient, safe, and easily producible carbon-based materials, integrating them with other antimicrobial agents and delivery systems. With continued optimization and clinical validation, carbon-based photothermal agents could become a vital tool in the fight against drug-resistant bacterial infections.

### 3.3 Polymer-based photothermal agents for treating bacterial infections

Polymer-based photothermal agents are composite materials formed by integrating polymer matrices with photothermally active substances, such as metallic nanoparticles, carbon nanomaterials, or organic dyes (Bayan et al., 2024). These polymers, which may be natural or synthetic, enhance the biocompatibility, stability, and functionality of photothermal agents (Fang Z. et al., 2023). Polymer-based materials not only improve the overall safety profile of PTT but also allow for controlled release and targeted delivery of photothermal agents, making them effective in clinical antimicrobial applications (Hu et al., 2024a).

Metallic nanoparticles like gold, silver, and copper exhibit excellent photothermal conversion efficiency and are often combined with polymer matrices to enhance their biocompatibility and antimicrobial efficacy (Li J. et al., 2024).

For example, AuNPs combined with PEG have demonstrated strong antimicrobial properties (Li J. et al., 2023). In one study, the gold nanoparticle-PEG composite effectively killed *MRSA* and *Escherichia coli* under NIR light (808 nm) irradiation, achieving a sterilization rate exceeding 95% (Ghayyem et al., 2022). The PEG coating enhances the stability of the AuNPs and minimizes their aggregation, making this composite material suitable for clinical applications such as wound dressings or implant coatings.

Similarly, AgNps show enhanced antimicrobial activity when combined with the naturally derived polymer chitosan (Luo Y. et al., 2022). This composite material has demonstrated potent bactericidal effects, particularly against multidrug-resistant bacteria like *MRSA* (Mei et al., 2024). Chitosan-based AgNp coatings have been applied to medical devices, such as urinary catheters and implants, to prevent bacterial infections and biofilm formation (Qi et al., 2022).

Carbon-based materials, such as CNTs and graphene, have high photothermal conversion efficiencies and, when integrated with polymer matrices, create multifunctional composites with potent antimicrobial properties (Ren et al., 2023).

For example, a composite material formed by combining CNTs with polypyrrole (PPy) has shown significant bactericidal effects against *MRSA* and *E. coli* under NIR light irradiation (Wang L. et al., 2023). Studies have indicated that the local temperature of this composite material reaches up to 60 °C upon light exposure, which is sufficient to damage bacterial cell membranes and cause cell death (Wang X. et al., 2024).

Graphene exhibits excellent antimicrobial activity when combined with chitosan, particularly in fighting biofilm-associated infections (Wu et al., 2023). The graphene-chitosan composite has demonstrated strong bactericidal effects against both Gram-positive and Gram-negative bacteria, with enhanced penetration and destruction of biofilms (Zhang et al., 2024).

Organic dyes, such as ICG and phthalocyanine dyes, are widely used in PTT due to their efficient light absorption and photothermal conversion properties (Zhao et al., 2023). When combined with polymer matrices, these dyes exhibit improved stability and enhanced antimicrobial effects, especially in deep tissue infections.

For instance, the combination of ICG with chitosan has been shown to effectively kill both Gram-positive and Gram-negative bacteria under NIR light irradiation (Ni et al., 2022). This composite material is particularly useful for treating deep-seated infections that are otherwise difficult to reach using conventional treatment methods (Zhao et al., 2023).

Similarly, zinc phthalocyanine combined with polyaniline has shown high photothermal conversion efficiency and has been successfully applied to the treatment of bacterial infections (Bayar et al., 2023). This combination of organic dyes with polymer matrices ensures greater targeting of infected areas and reduces potential damage to surrounding healthy tissues (Zhou et al., 2022).

Polymer-based photothermal agents offer significant advantages in antimicrobial therapy, particularly for biofilm-associated infections and multidrug-resistant bacteria. By combining polymers with nanomaterials like metallic nanoparticles, carbon-based materials, and organic dyes, these agents deliver targeted photothermal effects with improved biocompatibility and stability.

However, several challenges remain in translating polymer-based photothermal agents into clinical use. These challenges include limited light penetration, high production costs, and concerns about long-term safety. Further optimization of polymer-nanoparticle combinations and the development of more efficient, low-cost, and biodegradable polymer-based photothermal agents will be critical for expanding their clinical applications.

Future research should also focus on creating multifunctional composite materials that combine PDT with other antimicrobial mechanisms, such as drug delivery or immune modulation. By integrating intelligent delivery systems, researchers develop polymer-based photothermal agents that offer more precise and controllable treatment options, advancing their role in antimicrobial therapy.

## 4 Photothermal and photodynamic synergistic therapy for fighting bacterial infections

PTT and PDT are two highly effective antimicrobial strategies that use different photosensitizers and mechanisms to kill bacteria (Bai et al., 2023). Combining these two therapies allows for synergistic effects, where the interaction between photothermal agents and photosensitizers enhances the antimicrobial efficacy, leading to an outcome greater than either therapy alone (Chen et al., 2022). This combination therapy not only kills bacteria more effectively but also overcomes some limitations of each individual method, such as PDT’s dependence on oxygen, making it particularly beneficial in hypoxic environments (Dong et al., 2023).

**FIGURE 5:**
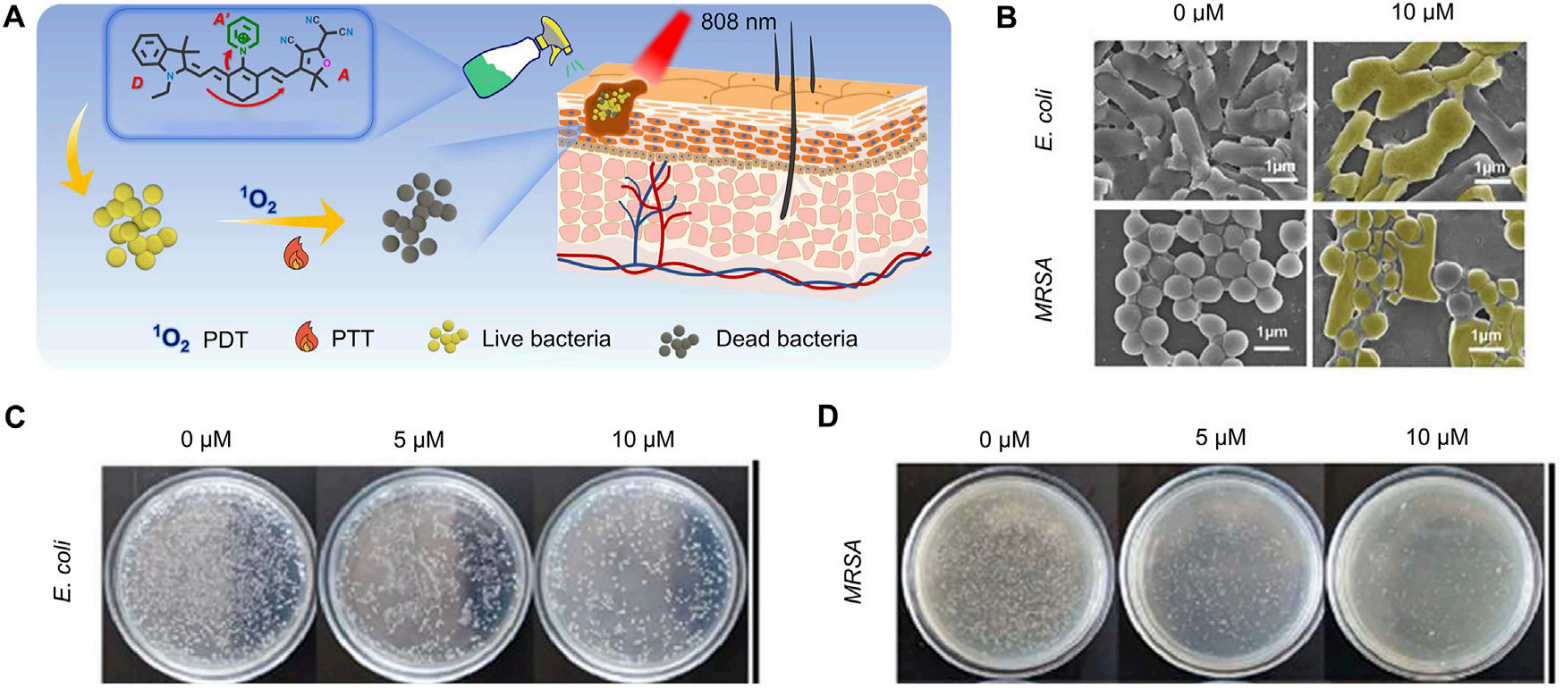
Antibacterial mechanism and effect of IND-Cy7 (Py)-TCF (Hao et al., 2024) **(A)** Schematic of the combined antimicrobial strategy **(B)** SEM images of bacteria treated with different concentrations of IND-Cy7 (Py)-TCF **(C)** Images of *Escherichia coli* AGAR plates treated with various concentrations of IND-Cy7 (Py)-TCF **(D)** Images of *MRSA* AGAR plates treated with different concentrations of IND-Cy7 (Py)-TCF. Copyright ^©^ 2024 Elsevier B.V.

Hao et al. developed an orthogonal molecular cationization strategy (IND-Cy7 (Py)-TCF) to enhance the ROS and thermal effects of Cy7 for the photodynamic and photothermal treatment of bacterial infections (Figure 5A) (Hao et al., 2024). IND-Cy7 (Py)-TCF was bactericidal in the dark against both *Escherichia coli* (∼10%) and *MRSA* (∼37%), probably due to the presence of a cationic Py moiety in the molecular structure. Even at low power (0.3 W/cm^2^), further irradiation with NIR light could increase the bactericidal effect to 50% against *Escherichia coli* and more than 80% against *MRSA* (Figures 5B–D).

The high temperature generated by PTT disrupts bacterial cell membranes, increasing their permeability (Ge et al., 2024). This allows ROS produced by PDT to penetrate bacterial cells more easily, further oxidizing bacterial lipids, proteins, and nucleic acids and leading to severe cell damage and death (Hu et al., 2024b). PTT also induces protein denaturation and coagulation, which is complemented by the oxidative damage caused by ROS produced by PDT, providing a powerful one-two punch against bacterial cells (Li et al., 2022).

In biofilm infections, PTT loosens the structure of the biofilm matrix, making it more permeable to photosensitizers (Liu B. et al., 2022). Once photosensitizers reach deeper into the biofilm, they produce ROS that synergistically kill the bacteria residing within the protective biofilm layer (Luo X. et al., 2022).

AuNPs are widely known for their excellent photothermal conversion ability, while porphyrin photosensitizers are effective producers of ROS under light irradiation (Shao et al., 2022). When combined, these materials exhibit an extremely high bactericidal efficiency. In one study, a gold nanoparticle-porphyrin complex achieved a sterilization rate of greater than 99.9% against *MRSA* under NIR light irradiation (Ullah et al., 2024; Wang Y. et al., 2024). This synergistic treatment not only destroys the bacterial cell membrane but also effectively eliminates biofilm-forming bacteria, making it a promising option for tackling drug-resistant infections (Wang J. et al., 2023).

CNTs have excellent photothermal conversion properties, and zinc phthalocyanine (ZnPc) is a commonly used photosensitizer in PDT (Liu et al., 2021). The combination of these two materials has shown remarkable results in combating biofilm infections (Wang J. et al., 2024). In one study, carbon nanotube-zinc phthalocyanine complexes demonstrated a sterilization rate greater than 97% against *Pseudomonas aeruginosa* biofilms under light irradiation. This synergistic effect not only provided efficient bacterial killing but also inhibited the formation and regeneration of biofilms, showing its great therapeutic potential in treating chronic and refractory infections (Wang P. et al., 2024).

The combination of AgNPs with rhodamine B photosensitizer demonstrates the potent synergistic effect of photothermal and photodynamic therapies under light exposure. In one study, the AgNps-rhodamine B complex achieved bactericidal rates of greater than 98% and 95% against *Escherichia coli* and *MRSA*, respectively. Additionally, this combination produced a rapid antimicrobial effect, effectively killing bacteria within just 5 minutes of light exposure. Such rapid response highlights the potential of this synergistic therapy in clinical settings, where fast-acting treatments are crucial (Wen et al., 2022).

Photothermal and photodynamic synergistic therapy offers significant advantages in antimicrobial applications, particularly against drug-resistant bacteria and biofilm-associated infections (Xu et al., 2024). The combination of photothermal agents and photosensitizers amplifies the bactericidal effect while addressing some of the limitations of the individual therapies, such as oxygen dependence and light penetration (Yan H. et al., 2023).

Despite these advantages, further research is needed to optimize the combination of these therapies (Xiang et al., 2023). Key areas for improvement include enhancing the targeting capabilities of photosensitizers and photothermal agents, developing more efficient light delivery systems, and ensuring that healthy tissues are minimally affected during treatment (Yan Z. et al., 2023). Another important consideration is the long-term safety and metabolic behavior of these materials *in vivo*, especially for clinical applications.

Future research should focus on developing smart delivery systems that precisely target infected tissues and reduce off-target effects. By fine-tuning the combination of photothermal and photodynamic therapies, researchers create a more effective, safe, and adaptable antimicrobial treatment strategy. Additionally, expanding the range of infections treated by synergistic therapies, particularly deep tissue and multidrug-resistant infections, could revolutionize the field of antimicrobial therapy.

## 5 Conclusions and prospects

Photosensitizer-based antimicrobial strategies, including PDT, PTT, and their synergistic combinations, have made significant progress in the fight against bacterial infections, especially in the context of multidrug-resistant bacterial and biofilm-related infections. These strategies offer a promising alternative to traditional antimicrobial methods by utilizing light-activated agents that are both highly efficient and selective and have a low likelihood of inducing drug resistance (Table 1).

**TABLE 1:** Antimicrobial strategy of phototherapy.

Antimicrobial strategies	Type of material	Microbial targets	Summary of results	Reference
Photosensitizer (PDT)	Cl-Hem	*Staphylococcus aureus*, *Streptococcus* epidermidis	Under 640 nm laser irradiation, 50 μg/mL Cl-Hem increased the killing rate of *Streptococcus* epidermidis by 73%. There is no significant antimicrobial effect in the absence of light	Atac et al. (2024)
	Metal porphyrin-based photosensitizer	Methicillin-resistant *Staphylococcus aureus* (MRSA), *Escherichia coli*	Under light conditions, the output of reactive oxygen species increased significantly, and the sterilization rate reached more than 90%, which had broad-spectrum antibacterial activity	Pujari et al. (2024)
Photothermal agent (PTT)	Gold nanoparticles (GNRs)	*Staphylococcus aureus*, *E. coli*	Under 808 nm light, the local temperature increased to 60°C, and the sterilization rate exceeded 95%. Enhanced effect when combined with antibiotics	Doveri et al. (2023)
	Graphene Oxide (GO)	*Pseudomonas aeruginosa*, *Staphylococcus aureus*	Rapid heating under light can destroy bacterial membranes, with a sterilization rate of more than 90%, and has the effect of resisting biofilm formation	Chao et al. (2023)
Combination therapy (PDT + PTT)	Molecular cationization strategy (IND-Cy7 (Py)-TCF)	*Escherichia coli* and MRSA	Combined with photodynamic and photothermal interactions, the effect of 1 + 1>2 is achieved under the irradiation of dual light sources, which has a significant bactericidal effect on drug-resistant bacteria and biofilm infection	Hao et al. (2024)
	Carbon nanotube-zinc phthalocyanine complex	*Pseudomonas aeruginosa*	The composites exhibited efficient bactericidal activity under double light, with a sterilization rate of more than 97%, which effectively inhibited biofilm regeneration	Wang et al. (2024e)
	Gold nanoparticle porphyrin complex	MRSA	Under near-infrared light, the bacterial structure is destroyed by the synergistic action of PDT and PTT, and the sterilization rate is more than 90%, and the effect on deep tissue infection is significant	Ullah et al. (2024), Wang et al. (2024c)

Photodynamic therapy, which uses photosensitizers to generate ROS under light exposure, has demonstrated strong broad-spectrum antibacterial effects. This technique is particularly effective against biofilm-associated infections and multidrug-resistant pathogens. Advances in metalloporphyrin-based, organic small molecules, polymer-based, and nanocomposite photosensitizers have shown significant therapeutic potential in both experimental and clinical settings.

PDT, on the other hand, eliminates bacteria through heat generated by photothermal agents under light exposure. This method is effective in hypoxic environments, such as biofilms or deep tissue infections, where the lack of oxygen limits the efficacy of PDT. Metallic nanomaterials, carbon-based materials, polymer-based materials, and organic molecular photothermal agents have all demonstrated potent antibacterial effects in PTT applications.

PTT uses photothermal agents to convert light energy into heat under light, resulting in local temperature increases above 40°C–60°C, destroying bacterial structures such as cell membranes, proteins and DNA through high temperatures. Specifically, high temperature can increase bacterial membrane permeability, protein denaturation and DNA damage, which eventually leads to bacterial death. PDT generates reactive oxygen species (ROS), such as singlet oxygen and hydroxyl radicals, under the activation of light by photosensitizers. These ROS have extremely strong oxidative capacity and can damage bacterial cell membranes, proteins, and genetic material. The generation of ROS can oxidise bacterial membrane lipids, oxidise proteins and cause DNA damage in a short time, eventually leading to bacterial death.

Both PTT and PDT have shown promising results in the treatment of methicillin-resistant *Staphylococcus aureus* (MRSA) and methicillin-sensitive *Staphylococcus aureus* (MSSA). PTT does not depend on chemical agents in response to high temperature and is therefore effective against both MRSA and MSSA, and is particularly important for MRSA. Antimicrobial resistance of MRSA does not impair the bactericidal efficacy of PTT, which usually shows a high bactericidal rate (>90%). For PDT, both MRSA and MSSA showed significant sensitivity to ROS generation, which effectively disrupted the cell membrane and DNA structure of both. The bactericidal effect of PDT on MSSA and MRSA was more significant, especially when combined with antibiotics. The key advantages of phototherapy-based antimicrobial strategies lie in their high efficiency, broad-spectrum activity, selectivity, and the low risk of promoting bacterial resistance. These methods fill critical gaps left by traditional antibiotics and chemical antimicrobial treatments, making them a burgeoning field of research.

However, while significant progress has been made, several challenges remain. PDT and PTT may bring some side effects when applied locally, especially at high doses or with repeated treatment. The high concentration of ROS produced by PDT can cause oxidative damage to healthy tissues, and this damage may trigger local tissue necrosis. PTT produces excessive temperature in the light, which similarly causes damage to the surrounding healthy tissue and triggers local necrosis. These injuries are largely dose-dependent, and this risk can be effectively reduced by controlling the light source intensity, exposure time, and agent concentration. Although PDT and PTT are local treatments with few systemic side effects, long-term or repeated treatment may pose a certain risk of cancer.

Developing new photosensitizers with improved light absorption properties, higher ROS generation efficiency, and better stability under physiological conditions is necessary. Furthermore, the specificity of photosensitizers needs to be enhanced to ensure that they target only infected tissues and cause minimal to no damage to healthy tissues. The use of nanocarriers and other targeting strategies plays a crucial role in improving the delivery of these agents. Combinations of PDT and PTT with other antimicrobial mechanisms, such as immune modulation or targeted drug delivery, should also be explored to further enhance therapeutic efficacy. Additionally, preclinical and clinical studies should be strengthened to evaluate the safety, long-term biocompatibility, and metabolic behavior of photosensitizers. This is especially important for applications in humans, where concerns about toxicity and off-target effects remain.

The future of photosensitizer-based antimicrobial strategies is promising. As photosensitizer designs continue to be optimized and nanotechnology becomes more integrated with these strategies, the potential for intelligent phototherapy systems that precisely target infections will grow. In the coming years, phototherapy-based antimicrobial strategies are expected to become one of the mainstream methods for preventing and treating clinical infections, providing a more effective and safer alternative to combat the global challenge of bacterial infections and antibiotic resistance.

With continued innovation in this field, photosensitizer-based technologies have the potential to make a substantial impact on global health by offering novel and powerful solutions to the pressing problem of bacterial drug resistance.
